# Quantification of the pathological response and fate in the lung and pleura of chrysotile in combination with fine particles compared to amosite-asbestos following short-term inhalation exposure

**DOI:** 10.3109/08958378.2011.575413

**Published:** 2011-06-03

**Authors:** DM Bernstein, RA Rogers, R Sepulveda, K Donaldson, D Schuler, S Gaering, P Kunzendorf, J Chevalier, SE Holm

**Affiliations:** 1 Consultant in Toxicology, Geneva, Switzerland; 2 Rogers Imaging Corporation, Needham, Massachusetts, USA; 3 University of Edinburgh, ELEGI Colt Laboratory, Edinburgh, Scotland; 4 Harlan Laboratories Ltd., Füllinsdorf, Switzerland; 5 GSA Gesellschaft für Schadstoffanalytik mbH, Ratingen, Germany; 6 Experimental Pathology Services AG, Muttenz/Basel, Switzerland; 7 Georgia-Pacific, LLC, Atlanta, Georgia, USA

**Keywords:** Chrysotile, amphibole, fine particles, inhalation, pathology, lung

## Abstract

The marked difference in biopersistence and pathological response between chrysotile and amphibole asbestos has been well documented. This study is unique in that it has examined a commercial chrysotile product that was used as a joint compound. The pathological response was quantified in the lung and translocation of fibers to and pathological response in the pleural cavity determined. This paper presents the final results from the study. Rats were exposed by inhalation 6 h/day for 5 days to a well-defined fiber aerosol. Subgroups were examined through 1 year. The translocation to and pathological response in the pleura was examined by scanning electron microscopy and confocal microscopy (CM) using noninvasive methods.The number and size of fibers was quantified using transmission electron microscopy and CM. This is the first study to use such techniques to characterize fiber translocation to and the response of the pleural cavity. Amosite fibers were found to remain partly or fully imbedded in the interstitial space through 1 year and quickly produced granulomas (0 days) and interstitial fibrosis (28 days). Amosite fibers were observed penetrating the visceral pleural wall and were found on the parietal pleural within 7 days postexposure with a concomitant inflammatory response seen by 14 days. Pleural fibrin deposition, fibrosis, and adhesions were observed, similar to that reported in humans in response to amphibole asbestos. No cellular or inflammatory response was observed in the lung or the pleural cavity in response to the chrysotile and sanded particles (CSP) exposure. These results provide confirmation of the important differences between CSP and amphibole asbestos.

## Introduction

The marked difference in biopersistence and pathological response between chrysotile and amphibole asbestos has been documented in studies from a range of sources (Bernstein & Hoskins, 2006). This study, however, is unique in that it has examined a commercial chrysotile product that was used as a joint compound. In addition, the pathological response was quantified by compartment in the lung and translocation of fibers to and pathological response in the pleural cavity determined. The interim results from the study were presented in Bernstein et al. (2010). This paper presents the final results from the study, including the details of the measurements in the pleural cavity.

This study was specifically designed to evaluate the pathological response and fiber distribution within the lung and pleural cavity. The difficulty of sampling the thin plural surfaces has been well documented. As summarized by Zocchi (2002), direct *in vitro* measurements of the biophysical properties of the pleura appear unreliable, because the mesothelium is very labile (Fentie et al., 1986; Peng et al., 1994; Agostoni, 1998), and the procedures required to obtain specimens are likely to compromise it (Agostoni, 1998). In order to minimize pleural sampling artifacts, two independent methods were developed for examining the translocation of fibers to the pleural cavity and any associated inflammatory response following exposure either to the chrysotile and sanded particulate (CSP) or to the amosite asbestos. These methods included examination of the diaphragm as a parietal pleural tissue and the in situ examination of the lungs and pleural space obtained from freeze-substituted tissue in deep-frozen rats.

The diaphragm was chosen as the parietal pleural tissue for examination because it can be quickly removed at necropsy with minimal alteration of the visceral lung surface. An area which included an important lymphatic drainage site (stomata) on the diaphragmatic surface was selected for examination of possible inflammatory response using scanning electron microscopy (SEM) and for the presence of fibers using confocal microscopy (CM).

In order to examine the visceral pleura environment, including the subpleural lung, the visceral pleural itself, and the pleural space, a noninvasive method for determining fiber location, size, inflammatory, and fibrotic response was used on rats, which were deep frozen immediately after killing.

In this study, to simulate the exposures encountered during the use of the product, the joint compound was applied and then dust released during sanding was collected. Sanding of the joint compound resulted in concomitant exposure to both chrysotile fibers (inherent within the joint compound) and joint compound particles. However, few fibers >20µm were present after sanding. Consequently, in order to fulfill the requirements of the protocol on which the exposure design was based (EUR 18748 EN, 1999; ILSI, 2005) (>100 f/cc longer than 20 µm; this length category being related to patho-genesis), chrysotile fibers were added to the sanded joint compound (CSP).

### CSP sample characteristics

The specification and preparation of the Ready-mix used to produce the sanded material has been described previously (Brorby et al., 2008; Bernstein et al., 2010). Extensive characterization including comparison of the bivariate size distribution was performed to confirm that the chrysotile used in the recreated formulation in this study (JM 7RF3 from the leffrey Mine in Quebec, Canada) closely matched that from a historical sample of the joint compound (Brorby et al., 2008). No historical Ready-mix formulations specified use of amphibole asbestos at any time.

To simulate typical usage of the joint compound, the recreated material was applied to pieces of drywall the ends of which were sealed with tape according to the instructions for the original material. The material was allowed to dry for at least 48 h and then sanded. Individual boards were sanded for 20–30 min. Four different boards were used to obtain a sufficient mass of material for these studies. The sanded material was collected in a large Ziploc bag and the bag was sent to the Research and Consulting Company Ltd. (RCC; currently known as Harlan Laboratories Ltd.), Füllinsdorf, Switzerland, where the inhalation exposures were performed.

Amosite used in this study was from the same batch as used in previous studies (Hesterberg et al., 1997, 1999a,b; McConnell et al., 1999; Rogers et al., 1999).

## Materials and methods

All studies were conducted by RCC Ltd. (Basel, Switzerland) according to the Swiss Ordinance relating to Good Laboratory Practice adopted 18 May 2005 [RS 813.112.1]. This Ordinance is based on the OECD Principles of Good Laboratory Practice, as revised in 1997 and adopted 26 November 1997 by the OECD Council [C (97)186/Final].

A pilot-study using both chrysotile alone and CSP was performed to assess the feasibility of a mixed exposure study. A few animals were exposed to each test material for 5 days and then examined for up to 3 days postexpo-sure. The results, reported by Bernstein et al. (2008), confirmed the feasibility of performing this mixed exposure study. Detailed description of the fiber exposure methods is presented in Bernstein et al. (2008).

The methodology used in the fiber exposure and the in-life phases of the study conforms to the guideline issued by the European Commission (ECB/TM/26 rev.7, 1999) with the following enhancements:

- The fiber evaluation was performed using an Analytical Scanning Transmission Electron Microscope with Energy Dispersive X-ray analysis (ASTEM-EDS) using an accelerating voltage of 80 kV and a magnification of at least 10,000×.
- The analytical part of the ISO 13794 method for the determination of asbestos in ambient air by the indirect-transfer transmission electron microscopy (TEM) procedure was used.
- The stopping rules for fiber counting included spe cific rules for four different length categories as fol lows: 100 fibers with a length < 5 µm, 200 fibers with a length between 5 and 20 µm, and 100 fibers with a length > 20 µm and 100 particles.

### Sample preparation

The JM 7RF3 grade 7 chrysotile sample was received by RCC Ltd. from Exponent, Inc. The fiber as received contained fiber bundles, which were too thick to be rat respirable. In order to separate the fiber bundles, the fiber was processed using a small-scale, table-top, Cyclotec 1093 Sample Mill (FOSS Tecator, Sweden). This is a low volume device, which opens the fiber bundles while obviating thermal degradation or contamination. Samples of a few milligrams of chrysotile were placed into the Cyclotec for a period of 1 min. This procedure was repeated three times for each sample to effectively open the bundles. No further processing was performed on the chrysotile. As presented in the results, the length distribution of the processed fibers in the aerosol was consistent with that of the pre-Cyclotec processed chrysotile sample.

Amosite used in this study was from the same batch as used in previous studies (Hesterberg et al., 1997, 1999a,b; McConnell et al., 1999; Rogers et al., 1999).

### Animal exposure

Acclimation: All animals were acclimatized to the restraint tubes and the inhalation exposure conditions by sham dosing over a period of 4 days of ∼ 1, 2, 4, and 6h on each successive day, respectively.

Three groups of rats were exposed for 6 h per day for 5 days to:

- Group 1: Filtered air alone (negative control group).
- Group 2: A fixed exposure level of well-characterized CSP.
- Group 3: A fixed exposure level of well-characterized amosite asbestos fibers.

Groups of 93 weanling (8- to 10-week-old) male rats (HanRcc: WIST(SPF), Harlan Laboratories Ltd. Laboratory Animal Services, 4414 Füllinsdorf/Switzerland) were exposed by inhalation in a flow-past nose-only exposure system for 6 h/day for a period of 5 consecutive days either to CSP (group 2) or to amosite asbestos (group 3). This system was derived from Cannon et al. (1983) and is different from conventional nose-only exposure systems in that fresh fiber aerosol is supplied to each animal individually and exhaled air is immediately exhausted. Schematic diagrams of each exposure systems used in the study were presented in Bernstein et al. (2008). In the negative control group (group 1), 56 rats were exposed in a similar fashion to filtered air passed through an unloaded aerosol generator. In group 2, additional commercial grade 7RF3 chrysotile (from the same batch used to reformulate the joint compound) was added to the sanded powder containing short fiber chrysotile to assure compliance with the fiber exposure specifications of the EC protocol (EUR 18748 EN., 1999). In groups 2 and 3, a fiber concentration higher than that required by the EC Protocol of 100 fibers with length L > 20 µm/cm^3^ was used in order to assure there was sufficient long fiber exposure. No amphibole (tremolite) fibers were detected in any of the analytical TEM with energy dispersive x-ray analysis (EDS) examinations.

For group 2 (CSP), two aerosols were generated using individual rotating brush aerosol generators. A fiber aerosol was generated from chrysotile fiber 7RF3 and a separate dust/fiber aerosol was generated from sanded material. The chrysotile fiber aerosol was passed through a 500-ml Pyrex glass cyclone to assist in the elimination of fiber bundles. The sanded powder aerosol was passed through a micronizing jet mill to reduce the particle size to be rat respirable. In-line ^63^Ni charge neutralizers were used to reduce the electrostatic charge to Boltzmann equilibrium. Following the charge neutralizers, the fiber and powder aerosols were mixed through a stainless steel Y-connection and then delivered directly into the nose-only flow-past exposure chamber.

For group 3, the aerosol of the amosite fiber was generated using a rotating brush aerosol generator followed by a ^63^Ni charge neutralizer to reduce the electrostatic charge on fibers to Boltzmann equilibrium. The aerosol was then delivered directly into the nose-only flow-past exposure chamber.

Control animals were exposed to filtered air passed through a separate brush-feed generator.

The aerosol mass was sampled for ∼ 5h during each exposure. Aerosol samples were collected on the appropriate filters in the vicinity of the animal's snout. Likewise, the temperature, relative humidity, and oxygen concentration were measured on atmosphere/aerosol samples collected directly from the delivery tube in the breathing zone of the animals. Also, in order to monitor and control the gravimetric concentration of the sanded powder aerosol alone, filter samples were also taken from a sampling outlet following the micronizing jet mill.

The methods for the gravimetric determination of aerosol concentrations; sampling of fiber number and size distribution of aerosol concentrations; particle size of dust aerosol; counting rules for the evaluation of aerosol and lung burden samples by TEM; and clinical examination and body weights have been presented in Bernstein et al. (2008 and 2010).

### Methods for determination of postexposure endpoints

Fiber lung burden and histopathology were initially analyzed immediately following the end of the 5^th^ day of exposure. This was termed day 0 of the nontreatment postexposure period.

Postexposure endpoints were developed in order to best answer the questions posed by this study. In the lung, these included:

- determination of the size and number of fibers in the lung in order to determine the biopersistence of the fibers,
- pathological response to the presence of fibers using histological examination, and
- confocal microscopic examination in order to determine the lung compartments in which the fibers are located and to visualize the juxtaposition of the fibers within the lung and any associated cellular response.

In the pleura, endpoints included:

- determination of the size and numb er of fib ers on the diaphragm as a representative parietal pleural tissue, and any associated pathological response as a function of time postexposure, and
- examination of the lung pleural/interface using frozen chest sections in order to examine noninvasively the translocation of fibers to the pleural cavity and any pathological response.

Table 1 summarizes the end points analyzed in subgroups of rats at each of the postexposure time points shown. The detailed specifications of these methods have been presented in Bernstein et al. (2010).

**Table 1:** Postexposure end points analyzed in subgroups of rats at each time points shown

Days after cessation of the 5-day exposure	Air control	CSP	Amosite asbestos
0	Fiber lung burden	Fiber lung burden	Fiber lung burden
	Lung histopathology	Lung histopathology	Lung histopathology
	Lung confocal microscopy	Lung confocal microscopy	Lung confocal microscopy
	Pleura–diaphragm	Pleura–diaphragm	Pleura–diaphragm
1	–	Fiber lung burden	Fiber lung burden
2	–	Fiber lung burden	Fiber lung burden
7	–	Fiber lung burden	Fiber lung burden
		Lung histopathology	Lung histopathology
		Lung confocal microscopy	Lung confocal microscopy
		Pleura–diaphragm	Pleura–diaphragm
14	Fiber lung burden	Fiber lung burden	Fiber lung burden
	Lung histopathology	Lung histopathology	Lung histopathology
	Lung confocal microscopy	Lung confocal microscopy	Lung confocal microscopy
	Pleura–diaphragm	Pleura–diaphragm	Pleura–diaphragm
30	Fiber lung burden	Fiber lung burden	Fiber lung burden
	Lung histopathology	Lung histopathology	Lung histopathology
	Lung confocal microscopy	Lung confocal microscopy	Lung confocal microscopy
	Pleura–diaphragm	Pleura–diaphragm	Pleura–diaphragm
90	Fiber lung burden	Fiber lung burden	Fiber lung burden
	Lung histopathology	Lung histopathology	Lung histopathology
	Lung confocal microscopy	Lung confocal microscopy	Lung confocal microscopy
	Pleura–diaphragm	Pleura–diaphragm	Pleura–diaphragm
181	Fiber lung burden	Fiber lung burden	Fiber lung burden
	Pleura–frozen chest sections	Pleura–frozen chest sections	Pleura–frozen chest sections
272	Fiber lung burden	Fiber lung burden	Fiber lung burden
	Pleura–frozen chest sections	Pleura–frozen chest sections	Pleura–frozen chest sections
363	Fiber lung burden	Fiber lung burden	Fiber lung burden
	Pleura–frozen chest sections	Pleura–frozen chest sections	Pleura–frozen chest sections

Two methods were used to perform this analysis.

### Diaphragm

A biopsy punch (10 mm diameter) was used to collect tissue discs of a uniform area of parietal pleural for microscopic analysis. Fields of view were randomly selected and stacks of 25 serial sections each were recorded. Five fields of view were recorded from each parietal pleural tissue specimen. The dimensions of voxels in the recorded volume were (x, y, and z dimensions, respectively) 0.17, 0.17, and 0.39 µm.

### SEM image collection and analysis

Parietal pleural tissue specimens prepared for SEM were brought into focus at 1000× magnification. Random fields of view were recorded from each tissue piece and SEM image data were analyzed for inflammatory cells and fiber profiles.

Reported are all fiber profiles in length classes beginning with ≥3 µm. The procedure was to locate parietal pleural areas observed on the diaphragm, collect a series of images, move at least two field widths, and repeat the process.

The number of fibers in each field of view was counted by a human operator who was looking for the characteristic bright points or lines, which indicated a reflective or refractile fiber. In instances where free ends of the fiber were observed, fiber length was recorded using three-dimensional measurement techniques. Fibers in the parietal pleura were categorized as occurring:

- in contact with the parietal surface of tissue, and
- within the parietal tissue.

### Inflammatory cells

Pleural macrophages are a normal constituent of the pleural space. Numerous free cells localized in pleural spaces would represent inflammatory cells at these time points. However, we determined that counting the number of free cells adherent to the parietal pleural surface did not produce a measurable observation. Therefore, we report instances of presence or absence in areas in which adherent cells appear to be accumulated. Groupings of more than three cells were considered to represent an inflammatory response.

## Results

### Validation of aerosol generation procedure

**Figure 1:**
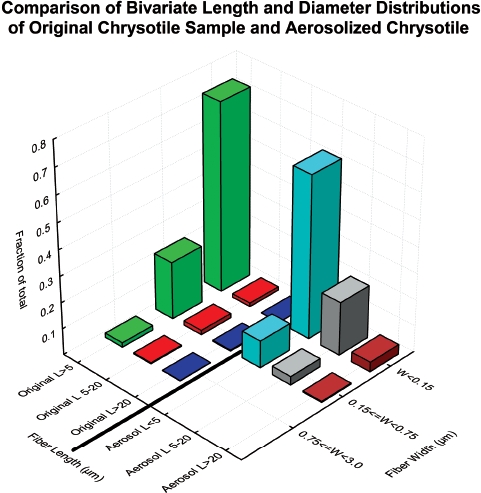
Comparison of the size distribution as determined by TEM of the original chrysotile sample (Brorby, personal communication) with that of the chrysotile aerosol to which the animals were exposed in this study. (See colour version of this figure online at http://www.informahealthcare.com/iht)

In this study, as described in the methods section above, the Grade 7 chrysotile fiber was prepared by passing it through a small-scale, table-top, Cyclotec 1093 Sample Mill (FOSS Tecator, Sweden). This is a low volume device, which separates the fiber bundles obviating thermal degradation or contamination. Brorby et al. (2008) reported on the TEM size distribution of the primary fibers and bundles of the Grade 7 chrysotile sample used in this study. Figure 1 compares the size distribution as determined by TEM of the original chrysotile sample (Brorby, personal communication) with that of the chrysotile aerosol to which the animals were exposed in this study. The methods used in this study successfully separated the thicker fiber bundles, removed the thicker fibers that were not respirable by the rat, and resulted in an aerosol exposure that was representative of the original material.

### Validation of lung digestion procedure

Comparative CM was used to assure that the lung digestion and TEM procedures used in this study did not affect the fiber dimensions of the chrysotile present in the lung (Bernstein et al., 2004).

The results of this analysis confirmed that there is a very good correlation between the length distribution as measured by the lung digestion procedure/TEM and the confocal methodology with a correlation *r*^2^ = 0.9. In addition, the TEM procedure does not reduce the length distribution of the fibers seen in confocal analysis. The mean number of fibers remaining at each time point for the chrysotile group showed a good correlation (*r*^2^ = 0.9) between the TEM measurements from the lung digestion procedure and the measurements obtained by CM.

The analytical examination of both the aerosol and the lung samples using TEM included routinely chemical identification of fiber type using energy dispersive x-ray analysis (EDS), which allowed also the detection of the crystalline structure of the fibers by selected area electron diffraction. No amphibole (tremolite) fibers were detected in the chrysotile samples in any of the TEM/ EDS examinations.

**Figure 2:**
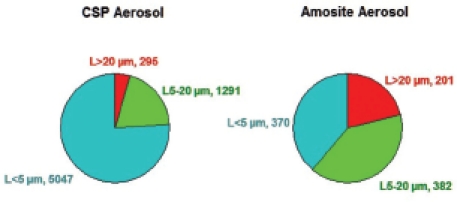
The mean number of fibers in the exposure atmospheres in each of the three length categories < 5 µm, 5–20 µm, and > 20 µm are illustrated for the CSP and amosite aerosols. (See colour version of this figure online at http://www.informahealthcare.com/iht)

The aerosol concentration and size distribution of all groups are shown in Table 2. The fiber aerosol concentrations were chosen based upon the EC protocol, which specifies that the exposure atmospheres should have at least 100 fibers/cm^3^ > 20 µm. In this study, additional chrysotile was added to the sanded material in order to achieve the mean number of fibers/cm^3^ > 20 µm. The resulting mean number of fibers per cm^3^ > 20 µm was 295 for chrysotile and 201 for amosite. Figure 2 shows the mean number of fibers in the exposure atmospheres in each of the three length categories < 5 µm, 5–20 µm, and > 20 µm for the chrysotile and amosite aerosols.

**Table 2:** Aerosol concentration and size distribution of the air control, CSP, and amosite inhalation exposure atmospheres

Exposure group	Gravimetric concentration mg/m^3^ (SD)	Density g/cm^3^	Number of fibers evaluated	Number of total fibers/cm^3^	WHO fibers/cm^3^	Percent WHO fibers	Number of fibers ≥ 20 µm/cm^3^	Percent of all fibers ≥ 20 µm/cm^3^	Diameter range (µm)	Length range (µm)	GMD (µm) (SD)	GML (µm) (SD)	Mean diameter (µm) (SD)	Mean length (µm) (SD)	Length weighted Arthm. Dmeter (µm)	Length weighted Geom. Ddiameter (µm)	Aspect ratio	Equivalent fiber diameter (µm)
(Group 1) Air control	0	—	2.0	0.078	0.042	53.8%	0	0%	n.a.	n.a.	n.a.	n.a.	n.a.	n.a.	n.a.	n.a.	n.a.	n.a.
(Group 2) CSP	2.64 (0.097)	2.6	2076	6543	1496	22.9%	295	4.5%	0.01–0.6	1.0–180	0.04 (2.26)	3.48 (2.33)	0.06 (0.07)	5.43 (8.93)	0.06	0.04	187.8	0.03
Sanded material	1.05 (0.085)	—	n.a.	n.a.	n.a.	n.a.	n.a.	n.a.	n.a.	n.a.	1.18 (MMAD)	n.a.	2.75	n.a.	n.a.	n.a.	n.a.	n.a.
(Group 3) Amosite	6.39 (1.19)	3.5	2059	953	584	61.2%	201	21.1%	0.03–1.2	0.8–150	0.26 (1.95)	8.13 (2.91)	0.32 (0.17)	13.81 (16.99)	0.39	0.29	50.9	0.17

The mean gravimetric concentration of the CSP exposure atmosphere was 2.64 mg/m^3^. The corresponding mean gravimetric concentration of the sanded material (including sanded 7RF3 chrysotile) was 1.05mg/m^3^, with the sanded material having a mass median aerodynamic diameter (MMAD) of 1.18 µm. The corresponding mean gravimetric concentration of the amosite exposure atmosphere was 6.39mg/m^3^. This gravimetric difference was due to the difference in the amosite fiber size distribution in comparison with the chrysotile.

The mean number of WHO fibers (defined as fibers > 5 µm long, < 3 µm wide, and with length :width ratios > 3:1; WHO, 1985) in the CSP atmosphere was 1496 fibers/cm^3^, which is > 10,000 times the OSHA occupational exposure limit of 0.1 fibers/cm^3^. The amosite exposure atmosphere had fewer shorter fibers, resulting in a mean of 584 WHO fibers/cm^3^. The mean total number of fibers of all sizes in the exposure atmosphere was 6543 fibers/cm^3^ for chrysotile and 953 fibers/cm^3^ for amosite.

The bivariate length and diameter size distributions of the CSP aerosol and the amosite asbestos aerosol have been presented in Bernstein et al. (2010).

### Fiber lung burdens

The mean concentrations and dimensions of the fibers recovered from the lungs at each time point for CSP and for amosite, respectively, are presented in Tables 3 and 4. For the CSP-exposed rats (Table 3), the mean number of fibers longer than 20 µm decreased from 0.31 million fibers L > 20 µm per lung immediately following exposure (day 0) to 0 fibers at 90 days. At day 0, fibers up to 90 µm in length were observed. The maximum fiber length steadily decreased from 7 days onward and from 90 days through 365 days, with the maximum length in the range of 20–25 µm. (At 181 days, one fiber of 21µm in length was observed on the filter aliquot analyzed from one animal and at 275 and 365 days one fiber of 25 µm in length was observed on the filter aliquot analyzed from one animal.) As these results were obtained through the lung digestion procedure, it was not possible to determine the association of these fibers with cells in particular macrophages. While in general the rat alveolar macrophage has been shown *in vitro* (Morimoto et al., 1994; Luoto et al., 1995; Zeidler-Erdely et al., 2006) to engulf fibers up to 20 µm in length, the results suggest that an occasional macrophage can engulf a slightly longer fiber.

**Table 3:** CSP-exposed lungs—mean concentrations and dimensions of the fibers recovered from the lungs at each time point

	0 Day	1 Day	2 Days	7 Days	14 Days	30 Days	90 Days	181 Days	275 Days	365 Days
	
B07031 Lung samples group 2—CSP	Mean	Mean	Mean	Mean	Mean	Mean	Mean	Mean	Mean	Mean
No. of fibers evaluated	2568.5	2471.0	2461.0	2339.0	2216.0	2098.0	1382.0	1252.5	1348.0	1940.0
No. of total fibers (millions/lung)/(standard deviation)	39.57 (11.73)	42.26 (9.64)	30.44 (5.32)	38.71 (3.21)	22.27 (6.00)	13.44 (3.11)	6.13 (0.67)	5.53 (0.42)	3.15 (0.76)	3.43 (0.79)
No. WHO fibers (millions/lung)/(standard deviation)	9.78 (2.79)	9.82 (2.02)	7.91 (2.75)	7.84 (3.21)	3.91 (1.65)	1.76 (0.54)	0.53 (0.17)	0.43 (0.10)	0.51 (0.28)	0.49 (0.03)
No. WHO fibers of total fibers (%)	24.7%	23.2%	26.0%	20.2%	17.5%	13.1%	8.7%	7.8%	16.3%	14.2%
No. of fibers L > 20 µm (millions/lung/(standard deviation)	0.31 (0.11)	0.24 (0.05)	0.23 (0.03)	0.11 (0.02)	0.02 (0.00)	0.01 (0.01)	0.00–	0.001-*	0.001–*	0.001–*
Fibers L > 20 µm of total fibers (%)	0.8%	0.6%	0.7%	0.3%	0.1%	0.1%	0.0%	0.0%	0.0%	0.0%
No. of fibers L 5–20 µm (millions/lung)/(standard deviation)	9.47 (2.70)	9.58 (1.98)	7.69 (2.74)	7.72 (2.59)	3.89 (1.65)	1.75 (0.54)	0.53 (0.17)	0.43 (0.10)	0.51 (0.28)	0.49 (0.03)
Fibers L 5–20 µm of total fibers (%)	23.9%	22.7%	25.2%	20.0%	17.4%	13.0%	8.7%	7.8%	16.2%	14.2%
No. of fibers L ≤ 5 µm (millions/lung)/(standard deviation)	29.79 (9.08)	32.44 (7.81)	22.53 (3.21)	30.88 (7.29)	18.37 (4.61)	11.68 (2.72)	5.60 (0.52)	5.10 (0.45)	2.64 (0.51)	2.94 (0.80)
Fibers L ≤ 5 µm of total fibers (%)	75.3%	76.8%	74.0%	79.8%	82.5%	86.9%	91.3%	92.2%	83.7%	85.8%
Diameter range (µm)	0.01–1.3	0.01–1.0	0.01–2.0	0.01–2.0	0.01–1.0	0.01–1.0	0.01–1.0	0.01–0.5	0.01–0.6	0.01–0.5
Length range (µm)	1.0.90	1.0.90	1.0.110	1.0.80	0.5.32	0.5.40	0.5.20	0.5.21.0	0.5.25.0	0.5.25.0
Mean diameter (µm)	0.11	0.09	0.10	0.10	0.09	0.07	0.06	0.06	0.05	0.04
SD	0.11	0.09	0.10	0.10	0.09	0.08	0.07	0.06	0.05	0.04
Mean length (µm)	4.60	4.45	4.66	4.02	3.55	3.19	2.83	2.95	3.69	3.59
SD	3.77	3.50	3.80	3.10	2.47	2.27	1.95	1.87	2.46	2.39
GMD (µm)	0.06	0.06	0.07	0.07	0.06	0.05	0.04	0.04	0.04	0.03
GSD	2.82	2.46	2.53	2.47	2.45	2.19	2.31	2.20	2.04	1.86
GML (µm)	3.72	3.64	3.82	3.30	2.95	2.59	2.31	2.49	3.07	2.99
GSD	1.88	1.85	1.84	1.84	1.93	1.98	1.93	1.80	1.90	1.85
Length weighted arithmetic dia. (µm)	0.13	0.11	0.12	0.12	0.10	0.09	0.08	0.07	0.07	0.05
Length weighted geometric dia. (µm)	0.07	0.07	0.07	0.07	0.06	0.06	0.05	0.05	0.05	0.04
Mode diameter (µm)	0.03	0.03	0.03	0.03	0.03	0.03	0.03	0.03	0.03	0.03
Mode length (µm)	2.5	2.0	2.0	2.0	2.0	2.0	2.0	1.5	2.0	2.5
Median diameter (µm)	0.05	0.05	0.05	0.05	0.05	0.03	0.03	0.03	0.03	0.03
Median length (µm)	4.0	4.0	4.0	3.5	3.0	2.5	2.5	2.5	3.0	3.0
Aspect ratio Mean	101.4	92.6	93.2	75.7	76.0	76.4	80.5	84.3	101.3	117.9
Total length of fibers per lung (m)	182	188 142	156 79	43 17	16.3	11.6	12.3			
Mass of fibers per lung in milligrams (density 2.6 g/cm^3^)	0.01	0.01	0.01	0.01	0.00	0.00	0.00	0.00	0.00	0.00
No. of particles evaluated	705.0	702.0	704.0	705.0	702.0	708.0	287.0	365.5	255.0	363.0
Mean no. of particles (millions/lung)	2.47	2.20	2.08	2.59	1.79	1.48	0.23	0.294	0.205	0.146
≤ 1 µm particles (millions/lung lobes)	2.06	2.08	1.98	2.46	1.75	1.48	0.23	0.292	0.205	0.145
> 1 µm to ≤ 3 µm particles (millions/lung lobes)	0.41	0.12	0.10	0.13	0.04	0.00	0.00	0.002	0.000	0.000
> 3 µm particles (millions/lung lobes)	0.00	0.00	0.00	0.00	0.00	0.00	0.00	0.000	0.000	0.000

* Standard deviation not presented as only one fiber was observed with length 21 µm—181 Days and 25 µm—272 and 365 days.

**Table 4:** Amosite-exposed lungs—mean concentrations and dimensions of the fibers recovered from the lungs at each time point

	0 Day	1 Day	2 Days	7 Days	14 Days	30 Days	90 Days	181 Days	275 Days	365 Days
	
B07031 Lung samples group 3—amosite	Mean	Mean	Mean	Mean	Mean	Mean	Mean	Mean	Mean	Mean
No. of fibers evaluated	2923.0	2883.0	2902.5	2881.5	2866.5	2860.5	2793.5	2811.0	2857.0	2844.5
No. of total fibers (millions/lung)/(standard deviation)	23.30 (8.04)	20.33 (2.79)	21.85 (5.41)	17.84 (4.62)	18.17 (4.65)	18.02 (3.13)	9.56 (3.15)	10.01 (1.99)	9.56 (0.93)	9.82 (1.25)
No. WHO fibers (millions/lung)/(standard deviation)	12.91 (4.02)	10.56 (1.48)	12.77 (3.21)	9.56 (3.00)	8.38 (2.03)	9.33 (1.54)	4.98 (1.68)	5.46 (1.68)	5.76 (0.93)	5.78 (0.96)
No. WHO fibers of total fibers (%)	55.4%	51.9%	58.4%	53.6%	46.1%	51.7%	52.1%	54.5%	60.2%	58.9%
No. of fibers L > 20 µm (millions/lung/(standard deviation)	2.74 (0.81)	2.24 (0.41)	2.81 (0.56)	1.98 (0.58)	1.63 (0.37)	1.98 (0.28)	1.10 (0.56)	1.11 (0.43)	1.33 (0.23)	1.39 (0.19)
Fibers L > 20 µm of total fibers (%)	11.8%	11.0%	12.9%	11.1%	8.9%	11.0%	11.5%	11.1%	14.0%	14.1%
No. of fibers L 5–20 µm (millions/lung)/(standard deviation)	10.16 (3.40)	8.32 (1.18)	9.96 (2.72)	7.58 (2.45)	6.76 (1.71)	7.34 (1.31)	3.88 (1.14)	4.35 (1.26)	4.42 (0.72)	4.39 (0.81)
Fibers L 5–20 µm of total fibers (%)	43.6%	40.9%	45.6%	42.5%	37.2%	40.7%	40.6%	43.4%	46.3%	44.7%
No. of fibers L ≤ 5 µm (millions/lung)/(standard deviation)	10.40 (4.31)	9.77 (2.24)	9.08 (2.41)	8.28 (2.90)	9.79 (3.24)	8.70 (2.33)	4.58 (1.57)	4.55 (0.42)	3.80 (0.48)	4.04 (0.55)
Fibers L ≤ 5 µm of total fibers (%)	44.6%	48.1%	41.6%	46.4%	53.9%	48.3%	47.9%	45.5%	39.8%	41.1%
Diameter range (µm)	0.03–3.0	0.03–3.0	0.02–3.0	0.02–2.0	0.03–1.5	0.03–1.5	0.03–1.6	0.03–1.4	0.03–2.0	0.03–1.5
Length range (µm)	0.5–110	0.6–110	0.7–100	0.6–125	0.8–90.0	0.6–105	1.0–110	0.5–105.0	0.7–95.0	1.0–105.0
Mean diameter (µm)	0.35	0.34	0.37	0.34	0.33	0.34	0.36	0.36	0.33	0.30
SD	0.21	0.22	0.26	0.19	0.19	0.18	0.21	0.19	0.19	0.18
Mean length (µm)	9.54	9.23	10.16	9.22	8.03	9.18	8.95	8.85	10.66	10.78
SD	10.11	10.16	10.68	10.10	9.01	9.76	9.78	9.37	10.71	11.59
GMD (µm)	0.29	0.28	0.29	0.27	0.27	0.29	0.29	0.30	0.27	0.23
GSD	1.84	1.99	2.06	2.02	2.00	1.85	2.11	1.98	2.11	2.19
GML (µm)	6.23	5.87	6.50	5.87	5.05	5.92	5.65	5.75	6.94	6.81
GSD	2.65	2.65	2.72	2.64	2.72	2.63	2.68	2.64	2.59	2.71
Length weighted arithmetic dia. (µm)	0.43	0.43	0.47	0.41	0.42	0.41	0.46	0.44	0.42	0.39
Length weighted geometric dia. (µm)	0.33	0.32	0.34	0.32	0.32	0.33	0.35	0.33	0.31	0.28
Mode diameter (µm)	0.40	0.40	0.20	0.40	0.20	0.40	0.30	0.35	0.35	0.35
Mode length (µm)	2.0	2.0	2.0	1.5	2.0	2.0	2.0	2.0	2.0	1.5
Median diameter (µm)	0.33	0.30	0.30	0.30	0.30	0.33	0.35	0.35	0.30	0.30
Median length (µm)	6.0	6.0	7.0	6.0	4.5	6.0	5.5	6.0	7.0	7.0
Aspect ratio mean	32.19	32.51	34.51	33.43	28.76	30.70	29.57	30.6	37.6	42.1
Total length of fibers per lung (m)	224.7	189.6	223.3	168.8	147.7	166.8	88.1	92.6	103.2	106.5
Mass of fibers per lung in milligrams (density 2.6 g/cm^3^)	0.15	0.13	0.19	0.10	0.09	0.10	0.06	0.06	0.06	0.06
No. of particles evaluated	357.0	297.5	201.5	317.5	379.0	317.0	465.5	287.0	201.0	163.0
Mean no. of particles (millions/lung)	1.38	0.93	0.79	0.87	0.85	0.87	0.72	0.440	0.369	0.316
≤ 1 µm particles (millions/lung lobes)	1.14	0.72	0.60	0.83	0.82	0.84	0.68	0.375	0.340	0.305
> 1 µm to ≤ 3 µm particles (millions/lung lobes)	0.23	0.19	0.19	0.04	0.02	0.02	0.04	0.065	0.029	0.012
> 3 µm particles (millions/lung lobes)	0.01	0.01	0.00	0.00	0.00	0.01	0.00	0.000	0.000	0.000

Clearance half-times of fibers.

For the amosite-exposed rats (Table 4), the number of fibers longer than 20 µm decreased slightly from 2.74 million fibers per lung immediately after exposure to 1.98 million fibers per lung at 7 days postexposure and 1.4 million fibers longer than 20 µm per lung observed at 365 days. The maximum fiber length observed 0 days postexposure was 110 µm and remained very similar throughout the 365 days postexposure period with a maximum length of 105 µm observed at 365 days.

**Figure 3:**
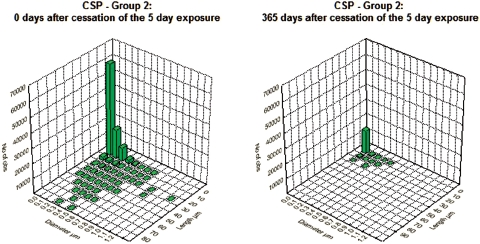
Bivariate length and diameter distribution of fibers in the CSP-exposed lungs, respectively, immediately after cessation of exposure (day 0) and at 365 days after cessation of exposure. (See colour version of this figure online at http://www.informahealthcare.com/iht)

**Figure 4:**
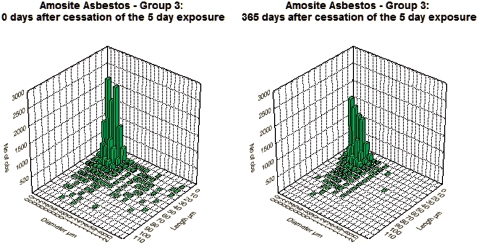
Bivariate length and diameter distribution of fibers in the amosite-exposed lungs, respectively, immediately after cessation of exposure (day 0) and at 365 days after cessation of exposure. (See colour version of this figure online at http://www.informahealthcare.com/iht)

Figures 3 and 4 show the bivariate length and diameter distribution of fibers in the CSP and the amosite-exposed lungs, respectively, immediately after cessation of exposure (day 0) and at 365 days after cessation of exposure. Only relatively short and thin fibers remain in the CSP-exposed lungs. For the amosite-exposed lungs, the thicker fibers, which likely deposited in the tracheo-bronchial tree, have been cleared by 365 days. The distribution of the thinner fibers remained remarkably similar to what was observed at 0 days postexposure.

**Figure 5:**
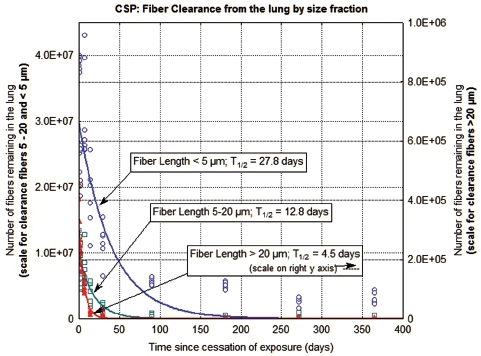
The clearance of fibers from the lung through 365 days postexposure is shown for CSP-exposed animals. The data and clearance curves for fibers < 5 µm in length, fibers 5–20 µm in length, and fibers > 20 µm in length are presented. (Note that the axis for the number of fibers remaining in the lung > 20 µm is on the right side of the graph.) (See colour version of this figure online at http://www.informahealthcare.com/iht)

**Figure 6:**
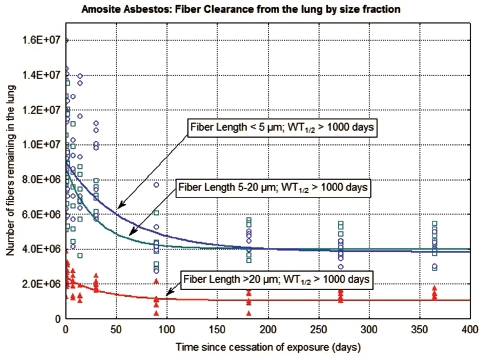
The clearance of fibers from the lung through 365 days postexposure is shown for amosite-exposed animals. The data and clearance curves for fibers < 5 µm in length, fibers 5–20 µm in length, and fibers > 20 µm in length are presented. (See colour version of this figure online at http://www.informahealthcare.com/iht)

The clearance of fibers from the lung through 365 days postexposure is shown in Figures 5 and 6 for the CSP-exposed animals and the amosite-exposed animals, respectively. Each figure shows the data and clearance curves for fibers < 5µm in length, fibers 5–20 µm in length, and fibers > 20 µm in length. Individual values for each animal and each size fraction are shown as are the clearance curves and the clearance half-times. For the CSP-exposed animals, the clearance curves were best fit using nonlinear estimation to a single exponential. For the amosite-exposed animals, the clearance curves were best fit using nonlinear estimation to a double exponential; with the clearance half-times expressed as the weighted T_1/2_ (EUR 18748 EN., 1999).

In the CSP-exposed animals (Figure 5), the fibers longer than 20 µm were rapidly cleared from the lung with a clearance half-time of 4.5 days. The fibers 5–20 µm in length were cleared with a half-time of 12.8 days, while the fibers < 5 µm in length cleared with a half-time of 27.8 days.

In the amosite-exposed animals (Figure 6), the number of fibers longer than 20 µm remaining in the lung showed a small reduction immediately following exposure with little subsequent clearance from the lung, with a weighted clearance half-time of > 1000 days. This initial reduction is likely due to fibers that deposited in the trachea-bronchial tree. The fibers 5–20 µm in length and the fibers < 5 µm in length also showed an initial reduction immediately following cessation of exposure. However, the strong inflammatory response created by the longer fibers appears to have locked-up the shorter fibers as well, with a weighted clearance half-time > 1000 days for these smaller length fractions.

### Histopathological results

The results from the histopathological examination of the lungs have been presented in detail in Bernstein et al. (2010). These findings confirmed that animals exposed to CPP produced no sign of pulmonary inflammation, aside from a macrophage response, at any time point. In contrast, the animals exposed to amosite asbestos showed a marked inflammatory response starting immediately after cessation of exposure (day 0). By 28 days postexposure, the lungs exhibited interstitial fibrosis with a Wagner Grade 4. These lesions were observed both in the conventional histopathological micrographs and in the 3D confocal micrographs. Because CM permits noninvasive imaging of a cube of tissue, micrographs using this technique were especially useful in showing the juxtaposition of especially the long amosite fibers in the tissue and their relationship to the observed lesions.

### Translocation of fibers to the pleural cavity and subsequent response

One important objective of this study was to examine the translocation of fibers to the pleural cavity using noneva-sive techniques and to evaluate the possible response to the fibers.

Two methods were used to perform this analysis. The examination of the diaphragm as a representative parietal pleural tissue was performed on the same animals that were examined for lung histopathology and CM. These animals were examined at time points starting at 0 days, immediately following cessation of exposure, through 90 days postexposure. The examination of the visceral pleural and the subvisceral pleural regions of the lung was performed on subgroups of animals starting at 181 days through 365 days postexposure. The visceral pleural examination method required immediate deep freezing of the rats following killing, which precluded other analyses and thereby accounted for the differential timing between the two methods.

### Diaphragm

The number of fibers in each field of view (average field of view was 0.03 mm^2^) was counted by a human operator, who was looking for the characteristic bright points or lines, which indicate a reflective or refractile fiber. In instances where free ends of the fiber were observed, fiber length was recorded using three-dimensional measurement techniques. Fibers in the parietal pleura were categorized as occurring:

- in contact with parietal surface of tissue, or
- within the parietal tissue.

### Inflammatory response to fibers

While pleural macrophages are a normal constituent of the pleural fluid (Noppen et al., 2000), the likelihood of finding individual macrophages adherent to the parietal pleural surface is small. Instead, examination was directed in areas in which adherent cells appear to be accumulated. Groupings of more than three cells (macrophages, neutrophils, etc.) were considered as representing an inflammatory response.

**Figure 7:**
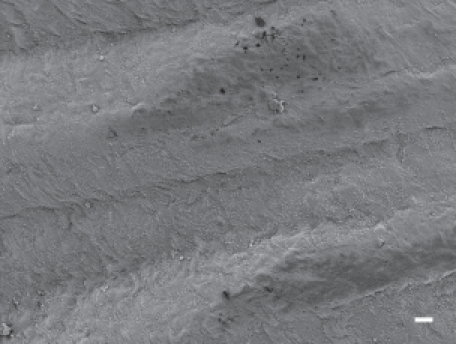
The parietal pleural surface from an animal exposed to CSP at 90 days postexposure. An occasional macrophage is observed; however, there is no associated inflammatory response or lesions.

In animals exposed to CSP, no parietal pleura lesions were observed at any time points studied. The parietal pleura surface from an animal exposed to CSP at 90 days postexposure is shown in Figure 7. An occasional macrophage is observed, however, there is no associated inflammatory response or lesions.

**Figure 8:**
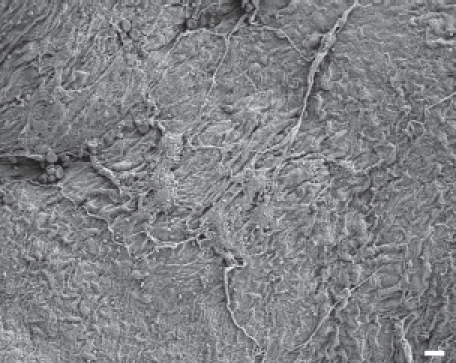
The parietal pleural surface from an animal exposed to amosite asbestos at 14 days postexposure. A network of large activated macrophages, which have laid down a fibrin matrix, is observed.

**Figure 9:**
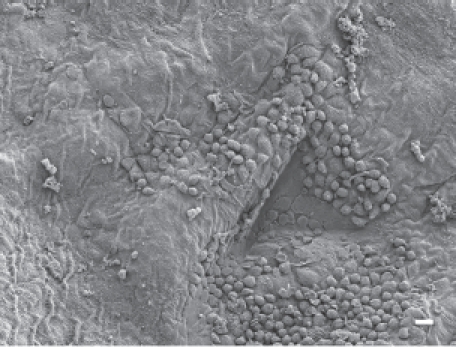
The parietal pleural surface from an animal exposed to amosite asbestos at 90 days postexposure. Numerous macrophages are observed on the parietal pleural (the triangular indentation seen in the micrograph was likely due to the back of a forceps, which was used for straightening the diaphragm after removal from the animal). Confocal imaging of the diaphragm indicated the presence of a number of amosite fibers in this region.

In animals exposed to amosite asbestos, when amosite fibers were observed on the parietal pleural surface, enlarged macrophages adherent to the parietal surface were also observed. Macrophages exhibited extended pseudopodia with Lamella project a, which is indicative of activated macrophages. Fibrotic lesions were also occasionally observed. As shown in Figure 8, a network of large activated macrophages and an associated fibrin matrix network was observed on the parietal pleural surface at 14 days postexposure. At 90 days postexposure, numerous macrophages were observed on the parietal pleural as shown in Figure 9 (The triangular indentation seen in the micrograph was likely due to the back of a forceps, which was used for straightening the diaphragm after removal from the animal). Confocal imaging of the diaphragm has indicated the presence of a number of amosite fibers in this region.

### Visceral pleural examination

While examination of the diagram provided a unique opportunity to examine the differential response on the parietal pleural surface, how fibers are transported to the pleural cavity and what impact they may have on the visceral pleural barrier have long been an open question.

To address this issue, the visceral pleural was systematically examined from cross-sections of rats that were frozen in liquid nitrogen immediately following sacrifice. This procedure was used in order to avoid possible artifacts that could stem from cross-contamination of fibers from the lung to the pleural cavity when tissues are manipulated at necropsy. The examination included a systematic survey using CM of the visceral pleural wall, the adjacent subpleural alveoli and the pleural space. The features of the tissues were evaluated and the location and length of any fibers present were determined.

**Figure 10:**
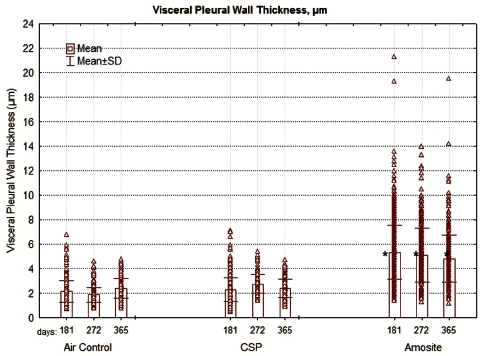
The thickness ofthepleural wall measured at between 5 and 10 points in each section examined is shown for the air control, CSP, and amosite asbestos-exposed groups. The mean visceral wall thickness of the amosite-exposed group was statistically larger than the mean visceral wall thickness of the chrysotile or air-control groups (Dunnett's *T*-test, *P* < 0.01).

In addition, the thickness of the pleural wall was measured at between 5 and 10 points in each section examined. The results of this analysis are shown in Figure 10. The visceral pleural wall averaged ∼ 2 µm in thickness in the air control group. The width ranged from < 1 µm to ∼ 7 µm and remained relatively constant from 181 days through 365 days postexposure. The visceral pleural thickness in the CSP-exposed rats was nearly identical to that of the air control animals with no statistical difference at any time point.

In the amosite-exposed rats, the mean visceral pleural thickness was ∼ 5 µm, more than double that of the air control and CPS exposure groups. This difference was statistically significant at all-time points from 181 to 365 days (Dunnett's t-test, p < 0.01). The thickness of the visceral pleural in the amosite-exposed animals ranged from ∼ 1 µm to > 20 µm.

**Figure 11:**
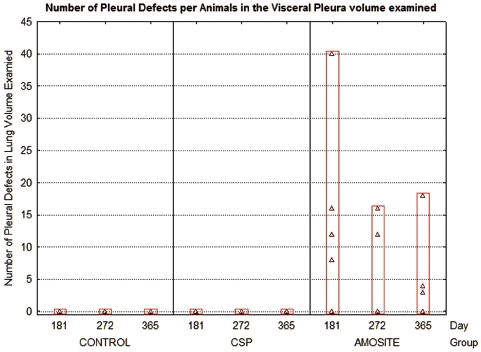
The average number of pleural defects per field of view (average field of view was 0.0075 mm^2^) is shown for the air control, CSP, and amosite asbestos-exposed groups.

Another measure of effect was the determination of pleural defects. Pleural defects were defined as a change in the pleural surface (visceral or parietal) thickness or surface interface appearance as indicated by connective tissue increase, accumulation of cells, or, in the case of visceral pleural side, appearance of subpleural alveolar involvement by inflammatory cells or connective tissue. The average number of pleural defects per field of view (average field of view was 0.0075 mm^2^) is shown in Figure 11. No pleural defects were observed in either the air control or the CSP-exposed animals at any time point. In the amosite-exposed rats, the number of pleural defects per field of view ranged from 40 at 181 days to > 15 at 365 days.

**Figure 12:**
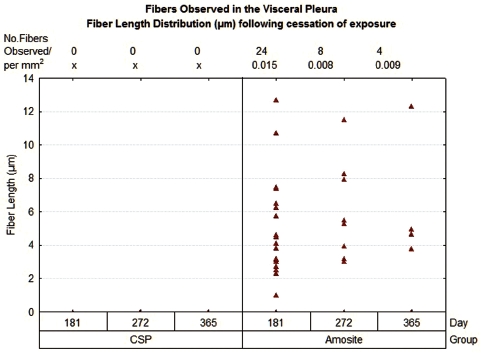
The number and length of fibers present at the visceral surface are shown.

The number and length of fibers present at the visceral surface were also quantified as shown in Figure 12. No fibers were observed at the visceral pleural surface in the air control or the CSP-exposed animals at 181, 275, or 365 days postexposure. In the amosite-exposed animals, an average of 16 fibers were observed at 181 days, 10 fibers at 272 days, and 4 fibers at 365 days normalized to a mean observed parietal pleural area of 0.01 mm^2^. The fiber length observed ranged from ∼ 1 µm to > 12.5 µm.

### Examination of the visceral pleura interface

#### Air control

**Figure 13:**
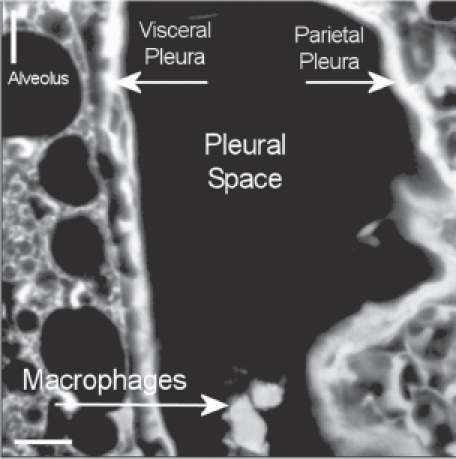
View of the pleural space at 181 days postexposure from a control animal is shown in the confocal image. The subpleural alveolar septa is seen on the left with the parietal pleura shown on the right. The brighter white is indicative of collagen in the visceral and parietal pleural walls. Free macrophages are present within the pleural space (bottom middle).

*A* typical view of the pleural space at 181 days postexposure from a control animal is shown in Figure 13. The subpleural alveolar septa is seen on the left with the parietal pleura shown on the right. The brighter white is indicative of collagen in the visceral and parietal pleural walls. Free macrophages are present within the pleural space (bottom middle).

**Figure 14:**
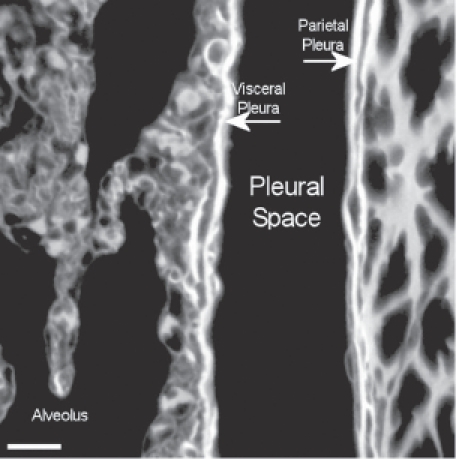
View of the pleural space at 272 days postexposure from a control animal is shown in the confocal image. The subpleural alveolar septa is seen on the left with the parietal pleura shown on the right. The brighter white is indicative of collagen in the visceral and parietal pleural walls.

The pleural space at 272 days postexposure from a control animal is very similar as shown in the confocal micrograph in Figure 14. The subpleural alveolar septa adjacent to visceral pleura are seen on the left, pleural space in the middle, and parietal pleura with the chest on the right.

#### CSP

**Figure 15:**
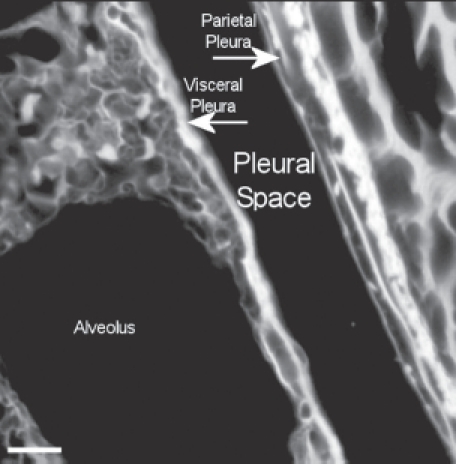
View of the pleural space from an animal exposed to CSP mixture at 272 days postexposure is shown in the confocal image. This confocal micrograph is very similar to that seen for the air control (Figure 14).

A typical field of view from an animal exposed to CSP mixture at 272 days postexposure is shown in Figure 15. This confocal micrograph is very similar to that seen for the air control (Figure 14).

#### Amosite asbestos

**Figure 16:**
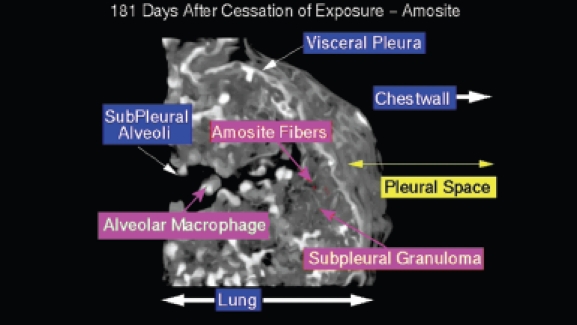
View of the pleural space from an animal exposed to amosite asbestos at 181 days postexposure. Amosite fibers are seen in a subpleural granuloma with numerous alveolar macrophages in the subpleural alveoli.

A confocal 3D micrograph of the visceral pleura and the adjacent subpleural alveoli from an animal exposed to amosite asbestos at 181 days postexposure is shown in Figure 16. Amosite fibers are seen in a subpleural granuloma with numerous alveolar macrophages in the subpleural alveoli.

**Figure 17:**
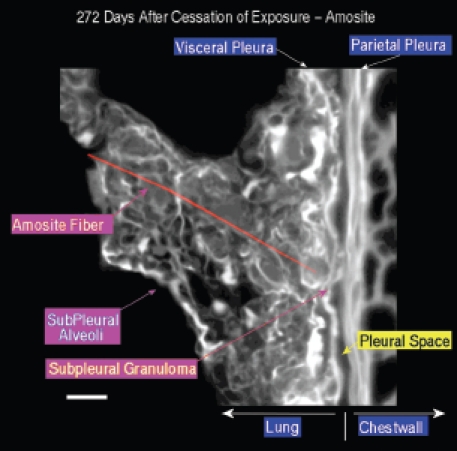
View of the pleural space from an animal exposed to amosite asbestos at 272 days postexposure. An amosite fiber ∼ 39 µm in length is observed within a subpleural granuloma. The right edge of this long fiber pierces the subpleural capsule. The thicker bright white matrix is indicative of a fibrotic thickening of the visceral pleura.

Figure 17 shows an amosite fiber ∼ 39 µm in length within a subpleural granuloma at 272 days postexposure. The right edge of this long fiber pierces the subpleural capsule. The thicker bright white matrix is indicative of a fibrotic thickening of the visceral pleura.

**Figure 18:**
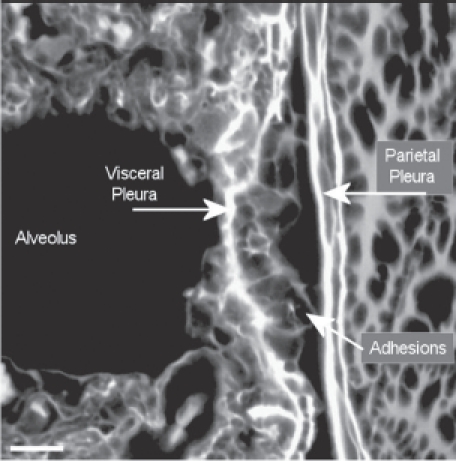
View of the pleural space from an animal exposed to amosite asbestos at 272 days postexposure. The subpleural alveolar septa seen in the left center of the image contains fibrotic lesions (thicker bright white matrix is indicative of enhanced collagen deposition). The parietal pleura and chest wall is shown on the right. Within the alveolus on the left, a number of subpleural macrophages can be seen.

A typical field of view from an animal exposed to amosite asbestos at 272 days postexposure is shown in Figure 18. The subpleural alveolar septa seen in the left center of the image contains fibrotic lesions (thicker bright white matrix, which is indicative of enhanced collagen deposition). The parietal pleura and chest wall is shown on the right. Within the alveolus on the left, a number of subpleural macrophages can be seen.

**Figure 19:**
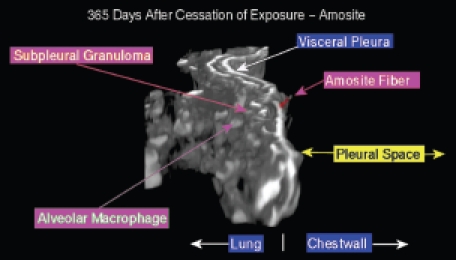
View of the pleural space from an animal exposed to amosite asbestos at 365 days postexposure. An amosite fiber penetrating the visceral pleural wall into the pleural space is seen. On the lung side, a well-developed subpleural granuloma is seen with alveolar macrophages on the surface.

Figure 19 shows an amosite fiber penetrating the visceral pleural wall into the pleural space at 365 days following cessation of exposure. On the lung side, a well-developed subpleural granuloma is seen with alveolar macrophages on the surface.

## Discussion

### Within the lung

As presented above, the exposure concentration of fibers longer than 20 µm/cm^3^ was more than double in the CSP-exposed rats as compared to the amosite-exposed rats and in both cases exceeded the number of fibers recommended in the protocol (ECB/TM/26 rev.7, 1999). Immediately following cessation of the 5-day exposure, the number of fibers > 20 µm in length remaining in the lung was 0.31 million for the CSP-exposed rats as compared to 2.74 million for the amosite-exposed rats. This is a result of the longer amosite fibers not dissolving or breaking apart in the lung in contrast to the chrysotile fibers, which rapidly break apart and are cleared.

### CSP

Chrysotile fibers are thin (0.8 angstroms) rolled sheets composed of magnesium silicate. The magnesium is on the outside of the sheet and can be solubilized in the alveolar surfactant. The crystalline structure of the silica matrix is attacked and broken apart by the acid environment of macrophages recruited in response to any inhaled particle (Pundsack, 1955; Wypych et al., 2005).

In this study, the CSP began clearing from the lung immediately following deposition, with a clearance half-time for fibers longer than 20 µm of 4.5 days. By the end of the 5-day exposure, more than 90% of the inhaled fibers longer than 20 µm had already been cleared from the lung (as compared to the amosite exposure in which the longer fibers were not cleared).

Once deposited in the lung, the longer chrysotile fibers quickly broke apart into shorter pieces. As reported earlier (Bernstein et al., 2010), fibers were initially deposited on the bronchial and alveolar surfaces with very few fibers found in the interstitial space. Those that were observed in the interstitial space were quickly removed and by 90 days postexposure, the remaining fibers were found only in the macrophages. No inflammatory or pathological response was associated at any time point with exposure to CSP.

The fibers between 5 and 20 µm in length had a clearance half-time of 12.8 days, and those fibers < 5 µm in length had a clearance half-time of 27.8 days. These slightly longer clearance half-times for the shorter fibers are probably due to the large number of fibers being created as the longer fibers break apart. These clearance half-times are still considerably less than those found for insoluble particles (Stoeber et al., 1970; Muhle et al., 1987). These values are similar to those presented in the interim result publication based upon the data through 90 days postexposure (Bernstein et al., 2010).

These results are comparable to those reported previously for pure chrysotile exposures (Bernstein et al., 2004, 2005a,b) and are consistent with the pilot study on CSP (Bernstein et al., 2008).

### Amosite asbestos

While CSP in the lung clears rapidly and produces no pathology, the amosite asbestos exposure group showed a markedly different response. Amosite has a notably different physical form than chrysotile. While chrysotile is a rolled thin sheet, amosite asbestos is a double-chain silicate formed as a solid cylinder of silica. Amosite has a very low dissolution coefficient even in an acid environment at environmental or human body temperatures. Amosite asbestos is biopersistent in both the lung and in the macrophage environments (Speil & Leineweber, 1969).

Following deposition in the lung, all fiber lengths of amosite persist with clearance half-times of > 1000 days, which is greater than the lifetime of the rat. For fiber lengths 5–20 µm and longer than 20 µm, these results are similar to the interim results presented in Bernstein et al. (2010) through 90 days postexposure. For the fibers < 5 µm in length, in the earlier publication, a clearance half-time of 90 days was estimated. However, incorporating the full set of data through 365 days, even the short fibers no longer clear after 90 days postexposure, most likely being locked up in the intense inflammatory response caused by the longer fibers.

Already by the end of the 5-day exposure, an intense inflammatory response to amosite was observed including granuloma formation around the longer fibers, which the macrophages could not clear. By 28 days postexposure, the continued inflammation resulted in the formation of interstitial fibrosis. The response to amosite is similar to that of another amphibole asbestos, tremolite, that has been studied previously (Bernstein et al., 2005b).

Immediately after the end of exposure, amosite fibers were observed penetrating the airway wall, located completely under the airway wall, as well as within macrophages on the surface of the ciliated epithelium. In the lung parenchyma, a smallnumber of fibers were observed partly or fully embedded into the interstitial space with fibers wholly or partly inside alveolar macrophages and touching alveoli, alveolar ducts, or respiratory bronchioles (Bernstein et al., 2010).

In contrast to the chrysotile fibers, at 90 days postexposure the amosite fibers were still observed penetrating the airway wall or located completely underneath the airway wall and on the surface of the ciliated epithelium. Even more important in terms of disease formation, substantial number of amosite fibers were found partly or fully embedded into the interstitial space with fibers observed wholly or partly inside alveolar macrophages and touching alveoli, alveolar ducts, or respiratory bronchioles (Bernstein et al., 2010).

The fibrotic response seen in this study following exposure to the amphibole amosite asbestos is similar to that reported in humans by Schneider et al. (2010). Schneider et al. (2010) reported that the fibrosis scores of the asbestosis cases correlated best with the number of uncoated commercial amphibole fibers.

### Translocation to the visceral pleura and then to the parietal pleura

#### CSP

Systematic examination of the region of the lung immediately adjacent to the visceral pleural and the visceral pleural itself using samples taken from frozen rats demonstrates no chrysotile fibers at any time point. In addition, no inflammatory cells or increase in collagen formation are observed at any time point, that is the tissue appears normal as it does in the negative control group. The thickness of the visceral pleural wall in the CSP-exposed animals was the same as that in the air control group.

Similarly, examination of the diaphragm as a representative parietal pleural tissue shows no indication of any inflammation at any time point in the CSP-exposed group. Unexpected interference was found in the signal from the confocal examination most likely due to protein coatings on the parietal pleural surface. As a result, the fiber examination can only be considered qualitative, not quantitative. In the CSP-exposed group, there was an indication of two possible shorter fibers present in one animal at 30 days postexposure. No other fibers were identified, and there was no cellular or inflammatory response associated with the two possible fibers.

#### Amosite asbestos

The range of pathological response observed in the amosite-exposed animals appears to mirror quite closely that which is observed in humans exposed to amphibole asbestos. Systematic examination of the region of the lung immediately adjacent to the visceral pleural and of the visceral pleural itself using samples taken in the frozen rat procedure has shown the presence of numerous amosite fibers as shown in Figures 16, 17, and 19. Figure 17 shows an amosite fiber ∼ 40 µm in length in the lung directly adjacent to the visceral pleural surface with one end piercing the visceral pleural capsule. Figure 19 shows an amosite fiber penetrating the visceral wall from the lung into the pleural space. An important inflammatory response with numerous macrophages and possibly other cells present is associated with these amosite fibers.

The confocal methodology used in this studyis quantitative in the assessment of excess collagen in the lung assayed by hydroxyproline (Antonini et al., 1999). The collagen is seen in the confocal micrographs as the bright white areas with the intensity of the brightness proportional to the amount of collagen. The normal lung as seen in the air controls in Figures 13 and 14 shows the normal collagen structure of the visceral and parietal walls as thin white lines on each side of the pleura. A nearly identical image seen in the CSP-exposed group is shown in Figure 15.

In the amosite-exposed group, as shown in Figure 17, both the visceral and the parietal pleural walls show a fibrotic response as indicated by the much brighter and thicker visceral and parietal surfaces on each side of the pleural space.

Adjacent to the end of the fiber (Figure 17), which is piercing the visceral pleural capsule, is a bridge to the parietal pleural surface, which is most likely an adhesion. In Figure 18, another example of adhesions between the visceral and parietal walls of the pleural cavity is seen in more detail with the folds of the adhesion appearing to extend out from the visceral surface to the parietal pleural surface.

The mean thickness of the visceral pleural wall in the amosite-exposed animals was more than twice that in the air control and the CSP. While the thickness of the air control and the CSP-exposed animals ranged up to 7 µm, that of the amosite-exposed animals ranged up to 21 µm. The greater thickness of the visceral wall in the amosite-exposed animals was associated with the presence of amosite fibers in these regions.

#### Amosite diaphragm

While the examination of the visceral pleural surface was performed in the frozen rat sections at intervals from 181 to 365 days postexposure, the examination of the parietal pleural surface (diaphragm) was performed at earlier intervals (between 0 and 90 days postexposure).

One of the most interesting results of this study is how quickly the amosite fibers reach the parietal pleural surface and initiate an inflammatory response. Within 7 days after the cessation of exposure, amosite fibers can be seen by CM on the diaphragm. By 14 days, an inflammatory response to these fibers was seen as shown in Figure 8. In addition, a fibrin matrix formed on the parietal pleural surface. Pleural injury and repair is characterized by disordered fibrin turnover, which contributes to the pathogenesis of pleural fibrosis. This is an early marker of pleural injury, and it has been proposed that disordered fibrin turnover plays a central role in the pathogenesis of pleural fibrosis. Fibrinogen is converted to fibrin forming the transitional intra-pleural neomatrix when intrapleural coagulation is activated by chemical or inflammatory stimuli (Jantz & Antony 2008; Shetty et al., 2008).

The formation of fibrin has also been associated with the development of adhesions between the visceral and parietal pleural walls (Idell et al., 2001) as shown in Figure 18. Intrapleural coagulation is initiated by increased local expression of tissue factors in response to the local injury and fibrinolysis is concurrently downregulated, due to primarily increased expression of PAI-1 and downstream antiplasmins. Formation of adhesions between the visceral and parietal pleural surfaces occurs in association with intrapleural coagulation and downregulation of fibrinolysis (Shetty et al., 2008). Pleural adhesions and fusion of the visceral and parietal pleura commonly occur with the diffuse pleural thickening (Huggins & Sahn, 2004), which was observed for amosite asbestos in this study (Figure 10).

In the quantification of the number of fibers in the CM examination of the visceral pleura, the area of the visceral pleural surface examined was recorded for each animal individually and ranged from a mean per time point examined of 0.0038–0.0076mm^2^. The total pleural surface area of the rat has been reported as 2450 mm^2^ (Bermudez et al., 2003), and approximately one-half of this would represent the visceral pleural surface. Based upon this and the number of fibers observed in these areas, and assuming that the distribution is uniform throughout the visceral pleura, the total fiber burden of the visceral pleura would be in the range of a few million fibers (all lengths).

## Conclusions

This study is unique in that it has examined a commercial joint compound product that historically used chrysotile and it quantified not only the pathological response and fiber distribution by compartment in the lung, but also the translocation of fibers to and pathological response in the pleural cavity.

The translocation to and pathological response in the pleura was examined by SEM and CM using noninvasive methods. The number and size of fibers was quantified using TEM and CM. This is the first study to use such techniques to characterize fiber translocation to and the response of the pleural cavity.

Amosite fibers were found to remain partly or fully imbedded in the interstitial space through 1 year postexposure and quickly produced granulomas (0 days) and interstitial fibrosis (28 days). Amosite fibers were observed penetrating the visceral pleural wall and were found on the parietal pleural within 7 days postexposure with a concomitant inflammatory response seen by 14 days postexposure. Pleural fibrin deposition, fibrosis, and adhesions were also observed in response to the amosite and were similar to that observed in humans in response to amphibole asbestos.

No cellular or inflammatory response was observed in the lung or the pleural cavity in response to the CSP exposure.

These results provide confirmation of the important differences between CSP compound and amphibole asbestos.
